# A nowcast model to predict outdoor flea activity in real time for the contiguous United States

**DOI:** 10.1186/s13071-023-06112-5

**Published:** 2024-01-22

**Authors:** Stella Self, Yuan Yang, Heather Walden, Michael J. Yabsley, Christopher McMahan, Brian H. Herrin

**Affiliations:** 1 Department of Epidemiology and Biostatistics, Arnold School of Public Health, University of South Carolina, Columbia, USA; 2 School of Mathematical and Statistical Sciences, Clemson University, Clemson, USA; 3 Department of Comparative, Diagnostic and Population Medicine, College of Veterinary Medicine, University of Florida, Gainesville, USA; 4 Southeastern Cooperative Wildlife Disease Study, Department of Population Health, College of Veterinary Medicine, University of Georgia, Athens, USA; 5 Warnell School of Forestry and Natural Resources, University of Georgia, Athens, USA; 6 Center for the Ecology of Infectious Diseases, University of Georgia, Athens, USA; 7 Department of Diagnostic Medicine/Pathobiology, College of Veterinary Medicine, Kansas State University, Manhattan, USA

**Keywords:** *Ctenocephalides*, Flea, Forecast, Mathematical model, Meteorological data

## Abstract

**Background:**

The cat flea (*Ctenocephalides felis),* a parasite commonly found on both dogs and cats, is a competent vector for several zoonotic pathogens, including *Dipylidium caninum* (tapeworms), *Bartonella henselae* (responsible for cat scratch disease) and *Rickettsia felis* (responsible for flea-borne spotted fever). Veterinarians recommend that both cats and dogs be routinely treated with medications to prevent flea infestation. Nevertheless, surveys suggest that nearly one third of pet owners do not routinely administer appropriate preventatives.

**Methods:**

A mathematical model based on weighted averaging over time is developed to predict outdoor flea activity from weather conditions for the contiguous United States. This ‘nowcast’ model can be updated in real time as weather conditions change and serves as an important tool for educating pet owners about the risks of flea-borne disease. We validate our model using Google Trends data for searches for the term ‘fleas.’ This Google Trends data serve as a proxy for true flea activity, as validating the model by collecting fleas over the entire USA is prohibitively costly and time-consuming.

**Results:**

The average correlation (*r*) between the nowcast outdoor flea activity predictions and the Google Trends data was moderate: 0.65, 0.70, 0.66, 0.71 and 0.63 for 2016, 2017, 2018, 2019 and 2020, respectively. However, there was substantial regional variation in performance, with the average correlation in the East South Atlantic states being 0.81 while the average correlation in the Mountain states was only 0.45. The nowcast predictions displayed strong seasonal and geographic patterns, with predicted activity generally being highest in the summer months.

**Conclusions:**

The nowcast model is a valuable tool by which to educate pet owners regarding the risk of fleas and flea-borne disease and the need to routinely administer flea preventatives. While it is ideal for domestic cats and dogs to on flea preventatives year-round, many pets remain vulnerable to flea infestation. Alerting pet owners to the local increased risk of flea activity during certain times of the year may motivate them to administer appropriate routine preventives.

**Graphical Abstract:**

**Figure:**
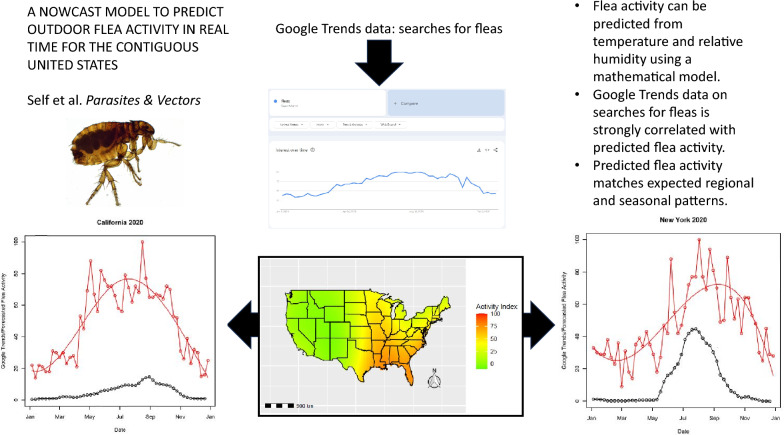

**Supplementary Information:**

The online version contains supplementary material available at 10.1186/s13071-023-06112-5.

## Background

*Ctenocephalides felis*, the cat flea, is the most common ectoparasite found on dogs and cats in North America and in many parts of the world [1, 2]. In addition to blood-feeding, cat fleas are known vectors of several pathogens, including *Dipylidium caninum*,* Bartonella henselae* and *Rickettsia felis*-like organisms (RFLO; i.e. *Rickettsia felis* and *R. asembonensis*) [1, 3]. Importantly, several of these pathogens are zoonotic. Flea allergy dermatitis is the most common dermatologic disease in dogs and cats and is caused by a hypersensitivity reaction to the saliva of the cat flea [4]. Because of the significant health risks to pets and humans, numerous topical and systemic products to control on-animal flea infestations are available, some of which can help control home infestations. However, previous studies examining pet owner behavior show that there are significant gaps in the purchase and administration of these preventives, which may be used on a seasonal basis [5]. Veterinary parasitologists recommend these products be given to all pets year-round, but exclusion of wildlife from raised patios and porches, reduction of pet off-leash activity and additional environmental treatment and management may be necessary at certain times of the year or in specific geographic regions where environmental conditions are best suited for flea development [4]. Understanding flea seasonality may help people working with outdoor hunting kennels, community animals, shelter animals and/or indoor-outdoor animals determine the specific times of the year to increase their flea preventive efforts. Management practices such as treating the shaded, protected microhabitats where fleas can develop with approved insecticides may become necessary at certain times, but the broad use of environmental pesticides is not recommended or warranted.

 The focus of the present study is to develop and validate a forecasting dynamic model to predict outdoor flea activity risk in the contiguous United States from environmental factors.

Fleas undergo a complete metamorphosis, with egg, larvae, pupae and adult stages, and all life stages persist in the environment until the adult finds a suitable host to feed on. Like many insects, the life-cycle of the cat flea can be altered by a variety of abiotic factors, including the presence of moist, shaded, protected microhabitats, environmental temperature and relative humidity (RH) [4]. Cat flea eggs and larvae have the highest survival rate between 13 °C and 35 °C and between 50% and 92% RH, with faster development time as temperatures increase [6]. Overall, the pupal stage is the most environmentally stable life stage, but the time to adult emergence also changes with temperature. Adults can emerge from the cocoon as soon as 12 days post-pupation at 27 °C but can take up to 155 days at 15 °C [7]. Temperature and humidity play a role at each life stage for both survival and developmental speed; therefore, efforts to predict flea exposure risk should include environmental data collected prior to and during adult emergence.

Previous research by Beugnet et al. created a mathematical model to describe the population dynamics of home flea infestations under a variety of conditions [8]. This work was expounded on in 2009 to create a matrix (recreated in Table 1) to describe the relative outdoor activity of fleas based on local temperature and humidity readings throughout France [9]. Here, activity is defined as the ability of fleas to develop from egg to adult, which would allow them to feed and reproduce. With the changing global climate, many researchers are investigating the changing distribution of arthropods, including *C. felis*. In Spain, models using regional environmental variables were combined with future climate projections to model distribution of the flea population and predict the spread of fleas throughout the country [10]. Similarly, prediction models of flea populations in Australia support the southward expansion of the flea populations as temperatures along the northern coast become unsuitable for flea development [11]. Species distribution modeling has been used with great success to predict the geographic distribution and relative abundance of several arthropods in addition to fleas, including ticks (*Ixodes* [12, 13] and *Dermacentor* spp. [14, 15]) and mosquitoes (*Aedes* [16, 17], *Anopheles* [18] and *Culex* [19, 20] spp.). Many of these tick and mosquito species are vectors of disease of serious public health concern and are thus subject to routine surveillance. Ongoing surveillance makes it possible to validate predictive models of the geographic distributions of these species. Unfortunately, flea surveillance is much more limited, making it difficult to validate predictions of flea abundance or habitat suitability.

**Table 1:** *Ctenocephalides felis felis* activity index table

Relative humidity	*Ctenocephalides felis felis* activity index^a^ at:						
	< 10 °C	10–15 °C	15–20 °C	20–25 °C	25–30 °C	30–35 °C	> 35 °C
< 40%	0	0	0	0	0	0	0
40–50%	0	0	0	10	10	0	0
50–60%	0	0	10	20	20	10	0
60–70%	0	0	30	40	40	20	0
70–80%	0	0	30	100	80	30	0
80–90%	0	0	40	100	100	50	10
> 90%	0	0	40	100	100	60	10

The table contains results from Beugnet et al. [9]

The activity index is a relative index of between 0 and 100 reflecting the ability of fleas to develop from egg to adult

In the absence of widespread flea surveillance efforts, predictive models of flea activity are difficult to validate. Given the large number of wildlife reservoirs for the cat flea, flea infestations on domestic dogs and cats would not provide an accurate account of flea populations. Gálvez et al. conducted flea surveillance on dogs across various bioclimatic zones in Spain, but they did not correlate their findings with the model created using the climatic variables [10]. In the USA, the flea surveillance data necessary to validate a flea activity forecasting model do not exist. In the absence of such data, we propose using Google Trends data as a surrogate for flea surveillance data. Automated data mining programs, like Google Trends, have become more frequently used to track public interest in specific topics across time and geographic region. This technique has been used to predict outbreaks of diseases like respiratory syncytial virus [21], monitor the introduction and spread of Chikungunya [22] and track the introduction of invasive animal species [23]. This method has also been used to track the seasonality and regionality of searches for ticks in comparison to the local temperature and humidity [24]. In this latter study, there was a correlation between the number of searches and favorable climate for tick activity that predictably varied throughout the year. Here, we present a flea forecast model for the USA and attempt to validate it using Google Trends data.

## Methods

### Forecast development

**Fig. 1:**
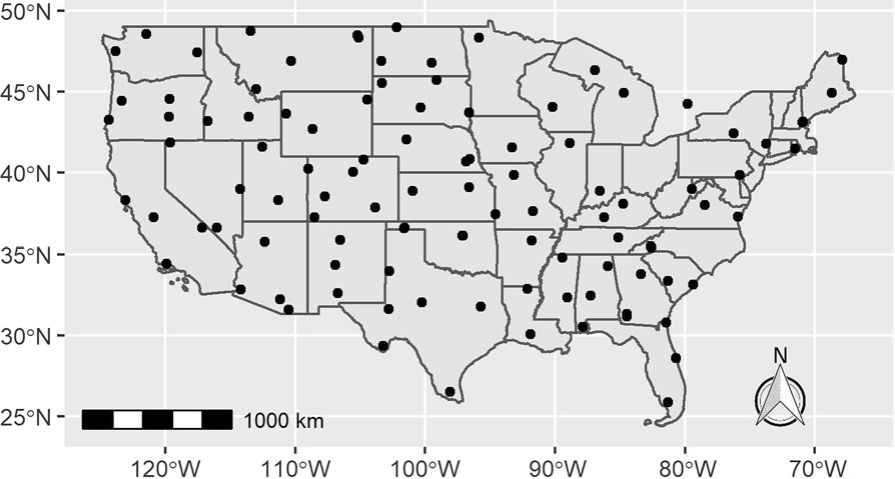
Sites for the 105 NOAA weather stations from which weather data were obtained to develop the nowcast flea activity forecast. NOAA, National Oceanic and Atmospheric Association

Our forecasting methodology is rooted in the work of Beugnet et al. who developed a mathematical model to predict outdoor flea activity for the cat flea based on temperature and RH [9]. For our study, weather data were obtained from 105 National Oceanic and Atmospheric Association (NOAA) weather stations (Fig. 1). Hourly data were obtained from each station from 1 January 2016 to 31 December 2020. Daily temperature and RH averages were then calculated by taking the arithmetic average of the 24 measurements for each day. For each station (*s*) and each day (*t*), the corresponding outdoor flea activity level $A(s,t)$ was computed from the daily average temperature and RH using Table 4 in Beugnet et al. [9], which is reproduced here as Table 1. These results are also based on research from Rust and Dryden [4] and Michel Franc’s PhD dissertation (National Veterinary School of Toulouse, Toulouse).

The aim of the forecast is to estimate outdoor flea activity for the upcoming week. Flea activity in the upcoming week depends not only on the weather conditions for that week, but also on past weather conditions. In optimal conditions, the flea life-cycle can be completed in approximately 21–30 days; consequently, the environmental conditions in the preceding 3 weeks are key to the survival and development of the larvae and pupae [4]. If conditions in the recent past have been unfavorable, then present activity will be lower due to decreased flea survival rates and slower developmental rates during the unfavorable period, resulting in fewer fleas. Our model incorporates the effect of past activity through weighted averaging. Specifically, the forecasted flea risk index at station* s* on day* t* is given by

$$F(s,t) = \sum_{i= -21}^{7}\frac{w(i)A(s,t+i)}{\sum_{j=-21}^{7}w(j)}$$

where $w\left(j\right)=\upphi \left(j/17.82\right)$ and $\upphi \left(x\right)$ denotes the probability density function of a standard normal random variable evaluated at* x*; the bandwidth of this Gaussian kernal is chosen that $w\left(-21\right)=w\left(0\right)/2$, that is, the activity from the present day receives twice as much weight as the activity from the most distant day. The forecasted flea risk index for a given location on day* t* is thus a weighted average of the outdoor flea activity from the past 21 days and the upcoming 7 days. Data from the upcoming 7 days are included to allow the model to forecast future outdoor flea activity. As weather forecasts are often not reliably available for more than 7 days into the future, only 7 days of future data are included.

The forecasted flea risk index map for day* t* was created by kriging the $F\left(s,t\right)$ values over all stations using a Gaussian covariance model. Kriging was performed using the krige function in the R Gstat package [25].

### Forecast validation

**Fig. 2:**
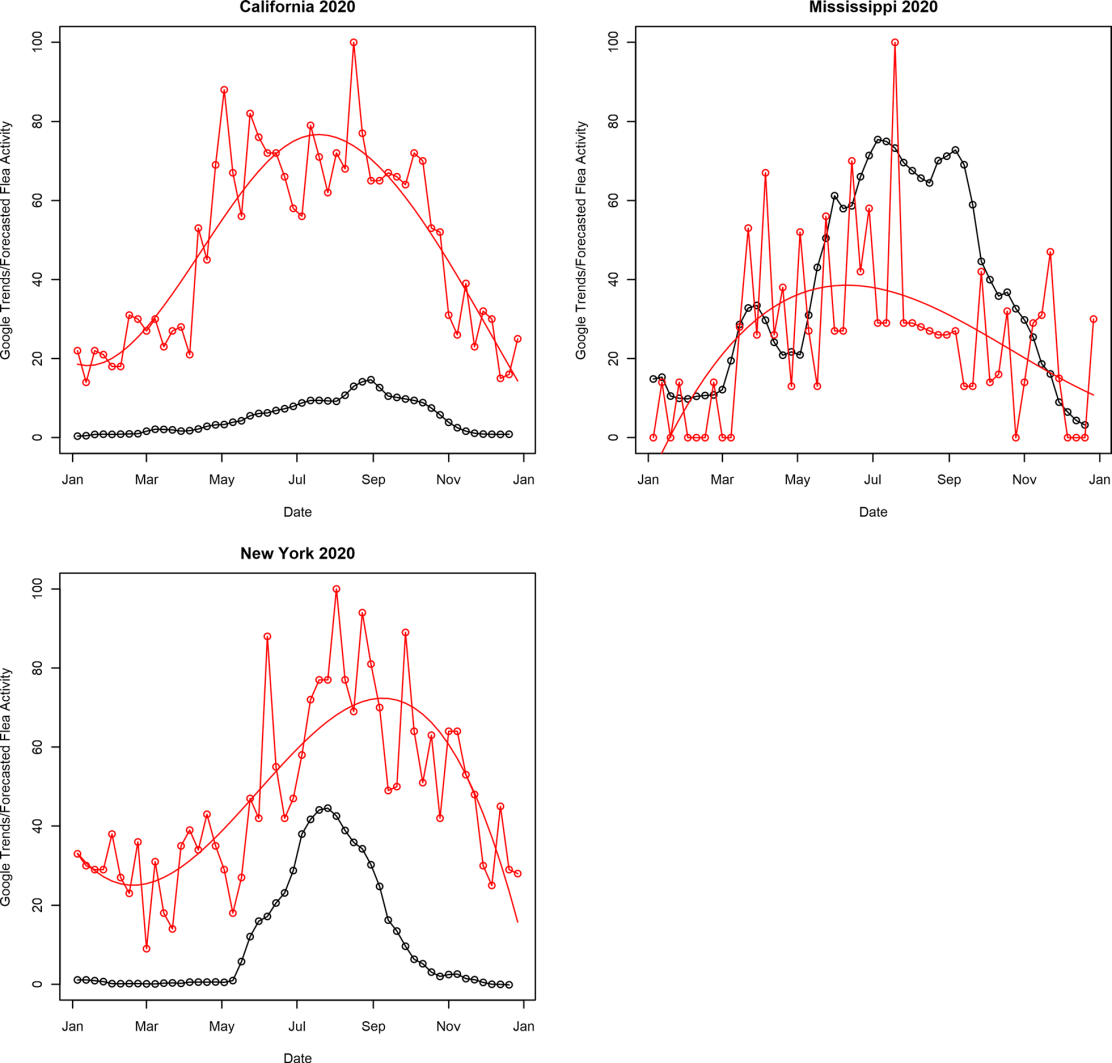
The aggregated forecasted flea risk index for 2020 (solid black line joining open circles), raw 2020 Google Trends data (solid red line joining open circles) and smoothed 2020 Google Trends data (red solid line) from California (top left), Mississippi (top right) and New York (bottom)

To validate the forecasted flea risk index, we used Google trends data. In particular, we used a geographically oriented metric reported by Google Trends that summarizes the number of searches for ‘fleas’ under the category ‘Pets and Animals’ as a surrogate for flea activity [26]. This metric provides a relative index of between 0 and 100 reflecting the number of Google searches for a specific topic conducted within a geographic region and time period. The index is relative to the geographic region and time period under consideration and does not allow for direct comparisons of the number of searches across regions. The Google Trends data used for our analysis were normalized using Google’s proprietary algorithm at the state and yearly level, which allows comparisons within the same year for the same state but does not allow for comparisons between different states in the same year or between different years in the same state. The proprietary algorithm rescales the number of searches in each state and year to produce an index of between 0 and 100 [27]. Since the degree of rescaling is different for each state and each year, one state can have a higher index than another without having more total searches. Similarly, the same state could have a higher index value in 1 year than another without having more searches in that year. This rescaling is necessary to account for differences in population among states and over time. However, *within* a given state and year, a higher index value does imply a higher number of searches. Georgia did not have Google Trends data available for all of the study years and was excluded from the validation analysis. For each of the remaining 47 contiguous US states and each year between 2016 and 2020, weekly trends data were obtained. This weekly data exhibited a considerable amount of volatility, particularly for states with smaller populations. Cubic B-splines were used to smooth the trends data. The spline model was fit to the trends data from each state-year separately with degrees of freedom ranging from 3 to 10. The degree of freedom resulting in the minimum Bayesian information criterion (BIC) was used to produce the final smoother for each state-year pair (see Fig. 2 for examples of the smoothed and unsmoothed trends data from California, Mississippi and New York from 2020).

To assess the correlation between the Google trends data and the forecasted flea risk index, the forecasted flea risk index was aggregated to the state and weekly level. The kriged daily forecasted flea risk was averaged over each state. These daily state-level averages were then averaged over each week (see Fig. 2 for example of the weekly averages for California, Mississippi and New York). For each state and each year, a linear regression model was then fit taking the weekly average flea forecast risk index from that state as the response and the smoothed Google Trends index as the predictor. The degree of correlation between the two datasets is assessed with correlation (*r*) and *R*^2^.

## Results

**Fig. 3:**
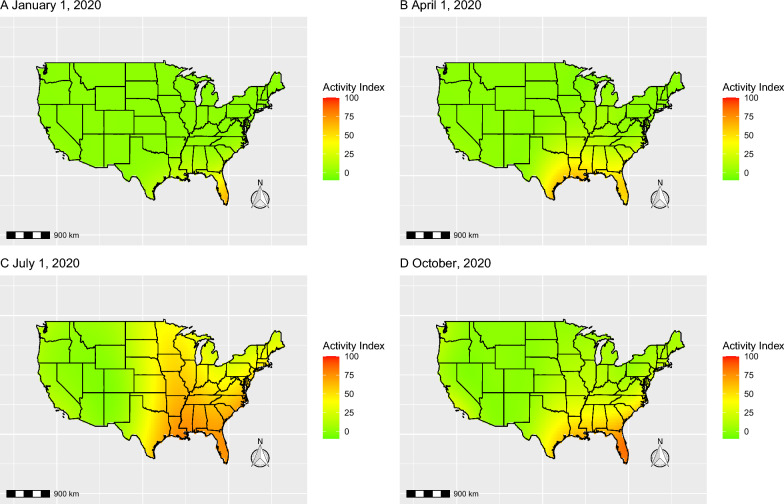
A selection of forecasted flea activity index values for four selected time periods: 1 January 2020 (**a**), 1 April 2020 (**b**), 1 July 2020 (**c**) and 1 October 2020 (**d**)]. The full video is available as Additional file 1: Video file

The forecasted outdoor flea activity maps for 2016–2020 are best visualized as a movie (Additional file 1: Video file); however, excepts from this video showing the forecast outdoor flea activity maps for 1 January 2020, 1 April 2020, 1 July 2020 and 1 October 2020 are included here (Fig. 3). During the spring months, activity begins to slowly increase throughout much of the southeastern USA, with higher activity levels spreading northward from the Gulf Coast (see Fig. 3b). During the summer months, activity is very high in the Southeast states and moderate in the Midwest and Northeast states (see Fig. 3c). By mid to late summer, there is also moderate activity on the Pacific Coast, although generally at lower levels than that seen in the Southeast states. Activity levels remain low in the Rocky Mountain and Southwest states throughout the year. During the fall, activity levels begin to subside in the Northeast and Southeast states, with the area of highest activity contracting back towards the Gulf Coast (see Fig. 3d). Similar trends are observed for the years 2016–2019.

**Fig. 4:**
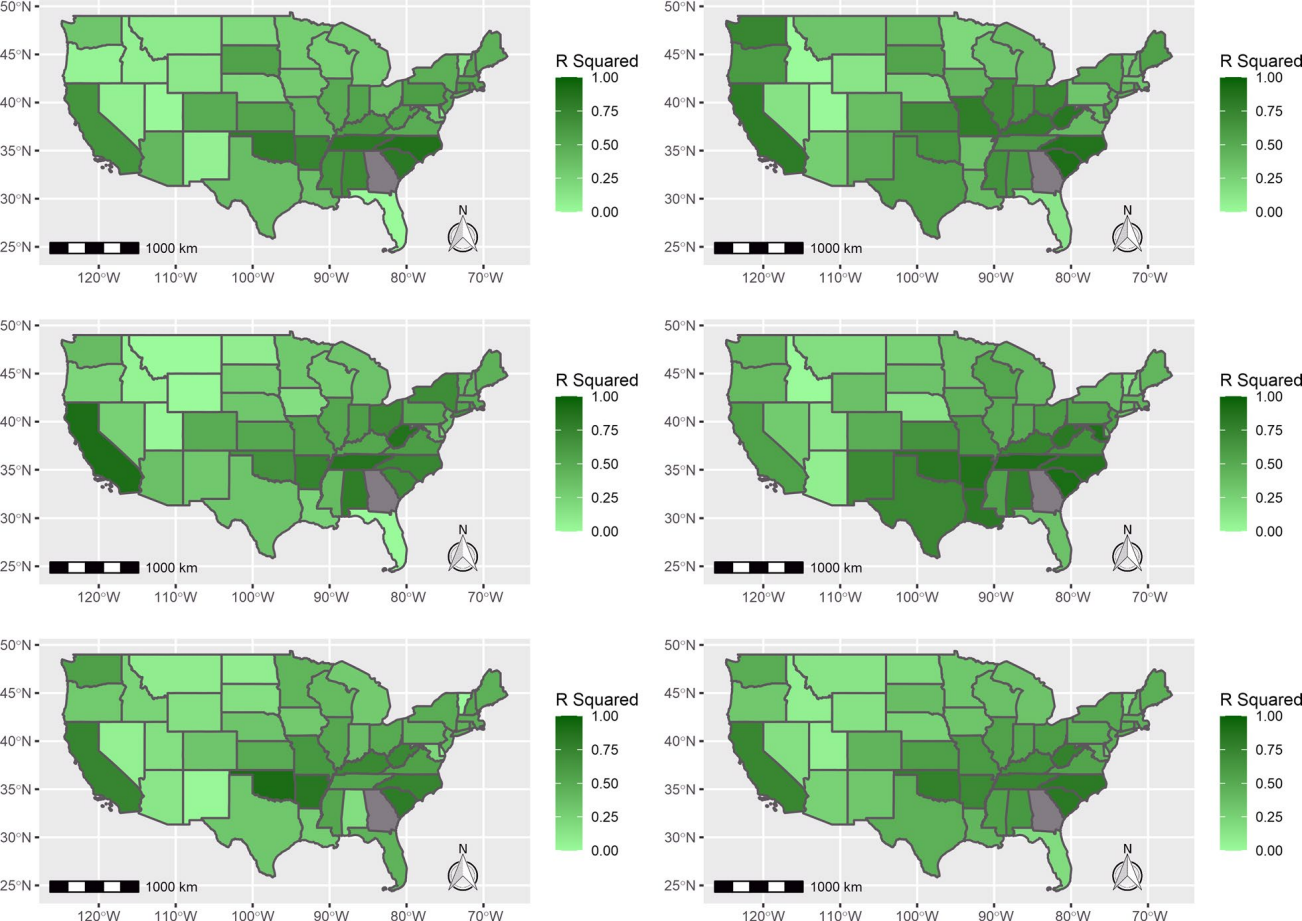
*R*^2^ values for each state from the linear regression model which regressed the weekly average forecasted flea activity index value on the smoothed Google Trends data for 2016 (top left), 2017 (top right), 2018 (middle left), 2019 (middle right) and 2020 (bottom left). The average *R*^2^ value across all 5 years for each state is shown in the bottom right. Google Trends data for Georgia were missing and therefore excluded from the analysis

The mean *R*^2^R values over all 47 states were 0.42, 0.49, 0.43, 0.51 and 0.40 for 2016, 2017, 2018, 2019 and 2020, respectively, indicating a moderate correlation between the forecasted outdoor flea activity level and the Google trends data (Fig. 4). These *R*^2^ values correspond to correlations (*r*) of 0.65, 0.70, 0.66, 0.71 and 0.63, respectively. The states with the highest *R*^2^ value in each year were North Carolina (2016: 0.88), South Carolina (2017: 0.90), California (2018: 90.1) South Carolina (2019: 0.92) and Oklahoma (2020: 0.93) (Fig. 4).

## Discussion

The forecasted outdoor flea activity video displays seasonal patterns where environmental conditions are favorable for flea development and emergence. The flea emergence cycles are predictable each year based on recurring climatic patterns, although yearly variations in the temperature and humidity affect the exact timing and duration of flea emergence.

The greatest year-round risk for fleas is in the far southern regions of the USA which stay warm and humid throughout the year. As seen in Fig. 3a, conditions in southern Florida remain hospitable for flea emergence throughout the winter, as has been previously reported [28]. To a lesser extent, these conditions exist along the Gulf Coast across to Texas and represent a risk for year-round flea infestations. By mid-spring, increasing temperatures create favorable environments throughout much of the Southeast USA extending up to Virginia (Fig. 3b). Some of the Upper Midwest states, like Illinois, will typically begin to notice infestations in habitats that are protected from direct environmental conditions, such as under raised homes or decks [29]. During the summer months, June through August, the risk for flea infestations peaks in the Upper Midwest and Northeast regions of the USA (Fig. 3c). While favorable conditions still exist in the South, the extreme heat and lack of humidity may reduce the amount of time the larval and adult flea stages can survive outside of shaded, protected microhabitats [29]. The July map (Fig. 3c) also highlights the regions with the lowest risk of *C. felis* infestations from the Southwest up the Rocky Mountain range to Montana. This region is too dry throughout the majority of the year to fully support the life-cycle of the cat flea [29]. Further west there is a slight increase in the risk for cat fleas in California up to Oregon. The temperate climates of the Central Valley of California and Pacific Northwest are conducive for flea emergence year-round, but the cooler temperatures may extend the length of time it takes to complete the full life-cycle [30]. During the fall, activity levels begin to subside in the Northeast and Southeast, with overall levels decreasing and the leading edge of highest activity moving southwards towards the Gulf Coast (Fig. 3d). This north-to-south shift as temperatures begin to drop leads many of the central states in the eastern half of the USA to experience a fall flea resurgence that can last from early October to late November depending on yearly conditions. The forecast maps indicate when environmental conditions are favorable for fleas to develop and survive, but cat fleas can survive harsh conditions while on wildlife or domestic hosts, in dens or burrows or inside/under buildings. This is especially true of pre-emerged adult fleas still residing in the cocoon, which are able to halt emergence for up to approximately 150 days until conditions are favorable for survival [7]. This allows cat fleas to continue their life-cycle throughout the year, posing a risk to cats and dogs year-round.

The southeastern states generally displayed higher* R*^2^ values than the rest of the country, with the states of Arkansas, North Carolina, South Carolina and Tennessee ranking in the top five highest * R*^2^ values for at least 3 of the 5 years. Less populous states tended to have lower * R*^2^ values. The states with the smallest * R*^2^ values changed from year to year, and there were 13 unique states which ranked in the bottom five in terms of * R*^2^ in one or more years. Of these 13 states, seven (Delaware, Idaho, Montana, Nebraska, North Dakota, Vermont, Wyoming) are among the 12 US states with fewer than two million people. Google Trends data are more reliable in states with larger populations, as the total volume of searches is higher in these areas. As a result, it is possible that the poorer performance in less populous states is due in part to the quality of the Google Trends data in these areas. Additionally, states that experience higher flea burdens, like those in the Southeast, had higher * R*^2^ values than those with lower flea burdens, like Montana and Wyoming. This can also affect the quality of the Google Trends data by selecting people who are more familiar with fleas and correctly searching for ways to control their infestations in states with high flea burdens, and also selecting people who are unfamiliar with fleas and incorrectly identifying pests within their home or on animals and searching for fleas.

Our model is subject to a few limitations. True seasonal data on flea populations are difficult to acquire. Very few comprehensive flea collection studies are being/have been conducted throughout the year in a specific state or region, let alone the entire USA. Many year-round studies focused on animal trapping or collecting fleas from animals brought to veterinary clinics [30, 31]. While these studies provide some local information, they do not describe the true risk of flea infestations as they are local, biased as to species investigated and based on on-host examinations, noting that on-host is only one of the ways that cat fleas survive harsh environmental conditions [7]. Therefore a reliable field method to validate forecasted flea models across the USA does not currently exist.

The spatial resolution of the weather data used to generate the flea forecasts is relatively coarse (Fig. 1). However, it has the advantage of consisting of direct measurements at weather stations as opposed to estimates from climate models. The spatial resolution of the Google Trends data (state level) is another limiting factor, as is the inability to meaningfully compare Google Trends data across states or years. As the correlation between the flea forecast and the Google Trends data is relatively low in some areas (particularly in less populous states), it would be desirable to have finer spatial resolution data to more closely examine the degree of non-concordance. Our model demonstrates the feasibility of predicting flea activity from weather data. The development and validation of a model using finer scaled weather data is an excellent area for future work. However, as the state-level Google Trends data are too spatially coarse to facilitate proper validation of a finer scale model, an alternate validation approach would be required.

Both the flea forecast and the Google Trends data are limited metrics which fail to fully capture flea activity. The flea forecast relies only on weather data and can (at best) only quantify *outdoor* flea activity. As indoor temperature and humidity usually differ substantially from outdoor conditions, the model’s ability to capture flea activity in domestic environments is quite limited. The model also fails to account for the effect of wildlife reservoir hosts, treatments and other flea prevention measures on flea activity. The Google Trends data are an imperfect proxy for flea activity and are subject to a number of potential biases. The number of searches for fleas could be influenced by many factors in addition to increased flea activity, including local television or social media discussion of fleas and/or pet owner perception of flea risk.

## Conclusions

In this work, we develop a ‘nowcast’ for outdoor flea activity levels derived from temperature and RH data. We then used it to create daily nowcast predictions for outdoor flea activity for the contiguous United States for 1 January 2016 to 31 December 2020. Finally, we validated our nowcast predictions using Google Trends data on searches for fleas. We found that our nowcast predictions are correlated with Google Trends data at the state level, suggesting substantial overlap between the times and places predicted to have high outdoor flea activity and the corresponding volume of flea-related searches. Furthermore, the seasonal patterns exhibited by the nowcast agree with local published studies on flea activity over the course of the year [1].

Despite the high degree of concordance between the nowcast predictions and the Google Trends data, the proposed nowcast model has several limitations. The model incorporates outdoor weather conditions only and does not account for indoor environments, protected microhabitats or on-animal reservoirs. Furthermore, the predicted outdoor flea activity index is a relative activity index and does not have a direct biological interpretation. Finally, Google Trends data are not a perfect surrogate for flea activity, as users may search for information on fleas for reasons other than a suspected flea infestation. Future work could assess the degree of correlation between flea activity derived from active surveillance and the predicted nowcast activity and/or the Google Trends data. While such surveillance would be infeasible on a national scale, it might be feasible to conduct surveillance on a well-defined subpopulation (e.g. fleas on feral cats in a particular county or state). Current real-time daily outdoor flea activity nowcasts produced from this model are freely available on the web at https://petdiseasealerts.org/flea-forecast-map/ by the Companion Animal Parasite Council.

### Supplementary Information

**Additional file 1: Video file.** Video file containing the daily flea activity forecasts from 1 January 2016 to 31 December 2020. Note: The video file must be opened in Adobe Acrobat.

## Data Availability

The data from this manuscript are publicly available at https://github.com/scwatson812/FleaForecast.

## Abbreviations

BIC: Bayesian information criterion
NOAA: National Oceanic and Atmospheric Association
RFLO: *Rickettsia felis*-like organisms
RH: Relative humidity
