# Supplementary material for: A nowcast model to predict outdoor flea activity in real time for the contiguous United States
Source: Parasit Vectors. 2024 Jan 22;17:27. doi: 10.1186/s13071-023-06112-5 (PMC10804753; doi:10.1186/s13071-023-06112-5)

2016−01−31

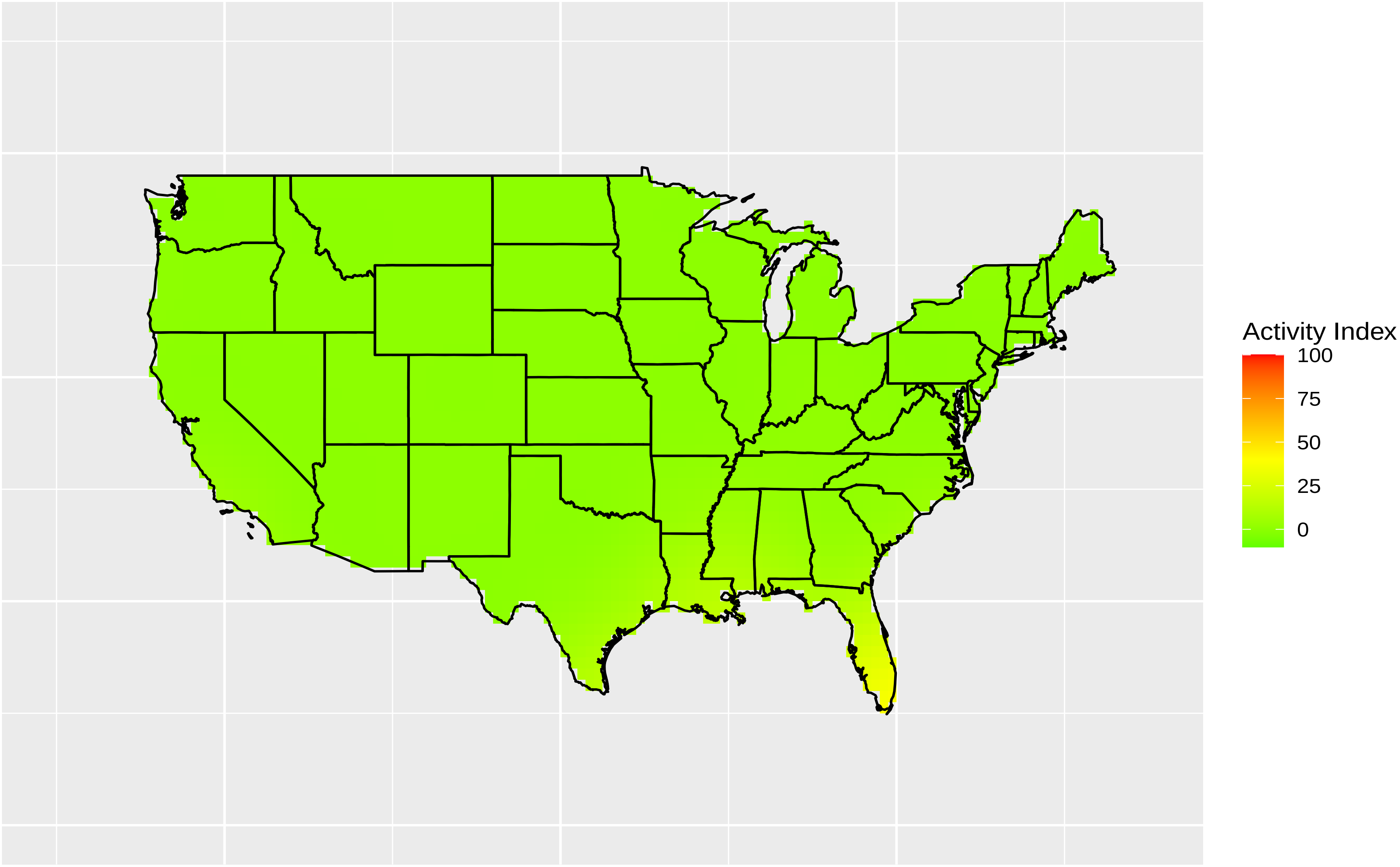

Supplement: Supplementary file 1 — Additional file 1: Video file. Video file containing the daily flea activity forecasts from 1 January 2016 to 31 December 2020. Note: The video file must be opened in Adobe Acrobat. [file 13071_2023_6112_MOESM1_ESM.pdf]
